# Dysregulated sphingolipid metabolism and autophagy in granulosa cells of women with endometriosis

**DOI:** 10.3389/fendo.2022.906570

**Published:** 2022-08-03

**Authors:** Bongkoch Turathum, Er-Meng Gao, Khwanthana Grataitong, Yu-Bing Liu, Ling Wang, Xue Dai, Ri-Cheng Chian

**Affiliations:** ^1^ Centre for Reproductive Medicine, Shanghai 10th People Hospital of Tongji University, Shanghai, China; ^2^ Department of Basic Medical Science, Faculty of Medicine Vajira Hospital, Navamindradhiraj University, Bangkok, Thailand; ^3^ Shanghai Clinical College, Anhui Medical University, Hefei, China

**Keywords:** mural granulosa cells, cumulus cells, metabolomes, sphingolipid metabolism, autophagy

## Abstract

We evaluated metabolic profiles between cumulus cells (CCs) and mural granulosa cells (MGCs) derived from women with endometriosis to identify their correlations with oocyte quality. CCs and MGCs were collected from women with and without endometriosis undergoing *in vitro* fertilization/intracytoplasmic sperm injection treatment. The metabolomics of CCs and MGCs were measured by liquid chromatography coupled to tandem mass spectrometry (LC-MS/MS) followed by a quantitative polymerase chain reaction to further confirm the genes involved in the metabolic results. LC-MS/MS analysis revealed differences in 24 metabolites of CCs and 71 metabolites of MGCs between groups. Among them, five metabolites were upregulated and 19 metabolites were downregulated in CCs with endometriosis, whereas three metabolites were upregulated and 68 metabolites were downregulated in MGCs with endometriosis. Metabolites related to sphingolipid metabolism, which included palmitic acid (PA) and docosahexaenoic acid, increased significantly only in CCs with endometriosis, whereas sphingosine and PA were significantly downregulated in MGCs with endometriosis compared with CCs and MGCs without endometriosis. Gene expression involved in ceramide synthesis (CERS1, SPTL1, and SMPD1) and autophagy (BECN1, LAMP, and PC3) were significantly higher in CCs with endometriosis according to FASN, BECN1, and LAMP protein expressions. However, gene expression involved in ceramide synthesis (SPHK1, ASAH1, and SGPP1) and autophagy (BECN1, LAMP, and PC3) were significantly lower in MGCs with endometriosis, whereas CERS1 and UGCG expression increased. There are differences in sphingolipid metabolites in CCs and MGCs with endometriosis compared with women without endometriosis. These differences seem to be involved in the regulation of autophagic cell death in preovulatory follicles.

## 1 Introduction

Endometriosis is an estrogen-dependent disorder affecting women of reproductive age and is characterized by the presence of endometrial tissue (glands and stroma) outside the uterine cavity and involved with chronic pelvic pain and infertility (1). Endometriosis affects fertility and several processes in the female reproductive system including folliculogenesis, ovulation, oocyte quality, and implantation (2). However, the mechanism of infertility is still unclear and insufficient to summarize the relation between endometriosis and its poor quality of oocytes (3).

Interestingly, recent studies demonstrated that endometriosis affects granulosa cells (GCs) in many aspects including an increased level of intracellular reactive oxygen species (ROS) generation, apoptosis, and dysregulation of the pathway involved in cell growth and development (4). During folliculogenesis, GCs play a critical role in the maturation of the oocyte. GCs differentiated into cumulus cells (CCs) and mural granulosa cells (MGCs) during the development of follicles. CCs are the cells in immediate contact with the oocyte, and MGCs line the follicular wall around the antrum. These cells play a critical role in follicular development and the maturation of oocytes (5).

Metabolomics is the new powerful tool for approaching disease progression *via* the new biomarker identification (6). Many reports demonstrated that metabolomic analysis in women with endometriosis in endometrial fluid (7), follicular fluid (8), urine (9), serum (10), and plasma (11) have represented the alteration of metabolite profiles. Palmitic acid (PA; 16:0) is the most common saturated fatty acid found in the human body (12) and can be provided the role of a precursor in sphingolipid metabolism-regulated several biological processes, including cell survival, migration, and apoptosis (13). Previous studies demonstrated that alteration of follicular free fatty acid (FFA) levels is involved with oocyte quality in both animals (14) and humans (15).

Recent studies also showed the excess palmitic acid-induced apoptosis of GCs in both animals (16) and humans (17), leading to impairment of ovarian follicular development and oocyte maturation. Moreover, it has been shown that sphingolipid metabolism changed in serum, peritoneal fluid, and endometrial tissue of women with endometriosis (13). In contrast, a report demonstrated that there is no specific metabolomic profile in the follicular fluid, which revealed no impairment of the cumulus–oocyte complex (COC) microenvironment of women with endometriosis (18).

In the present study, we aim to investigate the metabolic profiles in CCs and MGCs of women with and without endometriosis in order to find the correlation with the quality of the oocyte. We integrated the results of palmitic acid and sphingolipids’ balance to cell death at mRNA and protein levels *via* an autophagic pathway to evaluate the impact of endometriosis in the follicle related to the quality of the oocyte.

## 2 Materials and methods

### 2.1 Collection and isolation of cumulus cells and mural granulosa cells

After ovum pick up (OPU) by transvaginal ultrasound-guided needle aspiration, MGCs and CCs were collected from the follicular fluid under a stereomicroscope. The density gradient technique was used to purify MGCs and CCs by 40% and 80% gradient (SAGE), respectively. Then they were centrifuged for 15 min at 300 g, and the middle layer was collected. The remaining red blood cells were lysed by blood cell lysing buffer (Invitrogen by Thermo Fisher Scientific, Waltham, MA, USA). Then samples were washed by centrifugation 3 times with 1× phosphate-buffered saline (PBS). After that, cells were cryopreserved in liquid nitrogen until further metabolic analysis (19, 20).

### 2.2 Liquid chromatography coupled to tandem mass spectrometry

#### 2.2.1 Chromatography–mass spectrometry analysis

##### 2.2.1.1 Chromatographic conditions

CCs and MGCs were extracted by methanol/acetonitrile/aqueous solution (2:2:1, v/v) (21) and separated using Agilent 1290 Infinity LC ultra-high-performance liquid chromatography (UHPLC) hydrophilic interaction liquid chromatography (HILIC) column: column temperature was 25°C; flow rate was 0.3 ml/min. Mobile phase composition A = water +25 mM ammonium acetate +25 mM ammonia and B = acetonitrile. The gradient elution procedure was 0–0.5 min, 95% B; 0.5–7 min, linear change of B from 95% to 65%; 7–8 min, linear change of B from 65% to 40%; 8–9 min, B maintained at 40%; 9–9.1 min, linear change of B from 40% to 95%; and 9.1–12 min, B maintained at 95%. Samples during the entire analysis were placed in a 4°C autosampler (21, 22).

##### 2.2.1.2 Quadrupole time-of-flight mass spectrometry conditions

After the sample was tested, the first- and second-level spectra were collected using the AB Triple TOF 6600 mass spectrometer. The electrospray ionization (ESI) source conditions were carried out according to the instructions after HILIC separation. Ion Source Gas1 (Gas1) was set to 60, Gas2 was also 60, and Curtain gas (23) was 30. IonSapary Voltage Floating (ISVF) was ±5,500 V (positive and negative modes); source temperature was 600°C; the *m*/*z* range of time-of-flight (TOF) MS scan and product ion scan was 60–1,000 and 25–1,000 Da, respectively; the accumulation time of TOF MS scan and product ion scan were 0.20 s/spectra and 0.05 s/spectra, respectively; the secondary mass spectrum was obtained by information-dependent acquisition (IDA) and adopts high sensitivity mode; declustering potential (DP) was set to ±60 V (positive and negative modes); collision energy was 35 ± 15 eV. IDA setting excludes isotopes within 4 Da, with candidate ions to monitor per cycle of 6 (24–26).

### 2.3 Quantitative PCR

Total RNA was extracted by TIANGEN RNA simple Total RNA Kit (TIANGEN), and RNA concentration was measured by NanoDrop ND 2000 spectrophotometer (Thermo Scientific). qPCR was carried according to the manufacturer’s instructions: cDNA (1 μl), forward and reverse primers (0.2 μl, 10 μM), RNase-free ddH_2_O (3.6 μl), and ChamQ Universal SYBR qPCR Master Mix (5 μl). Three-step PCR amplification protocol was followed: 95°C 30 s; 95°C 10 s → 60°C 30 s, a total of 40 cycles; 95°C 15 s → 60°C 60 s → 95°C 15 s. The housekeeping gene GAPDH was used as an internal reference, and the specific primer sequences are shown in **Table 1** (27, 28).

**Table 1:** Gene-related information.

Protein	Gene	Size	Primer sequences (5′–3′)	Tm
Smase	*SMPD1*	162	F:5′-GCTGGCTCTATGAAGCGATGGC-3′	63.9
			R:5′-AGAGCCAGAAGTTCTCACGGGA-3′	63.1
SMSynthase	*SMS2*	144	F:5′-GCATTTCCAGTGTGCTCCAAAGC-3′	63.2
			R:5′-GTAACCGTGTGACCGCTGAAGA-3′	62.8
Serine palmitoyl transferase	*SPTLC1*	118	F:5′-GCAGTGTTGAAGGAAAAGTGCGG-3′	63.2
			R:5′-CAGTGCTCTCTTCCAGTTGTAGG-3′	60.6
Ceramide synthase	*CERS1*	90	F:5′-ACGCTACGCTATACATGGACAC-3′	60.3
			R:5′-AGGAGGAGACGATGAGGATGAG-3′	60.5
Dihydroceramide desaturase	*DEGS1*	519	F:5′-TTCTTCTGTACCGCTTTCAG-3′	55.4
			R:5′-TTACTCCAGCACCATCTCT-3′	55
Sphingosine kinase	*SPHK1*	51	F:5′-AGCTTCCTTGAACCATTATGCTG-3′	59.3
			R:5′-AGGTCTTCATTGGTGACCTGCT-3′	61.6
Ceramidase	*ASAH1*	149	F:5′-CTTTGCTGGCTATGTGGGCATG-3′	62.4
			R:5′-TGAGGAACCCTATCCACATGGC-3′	61.8
Ceramide synthase	*CERS1*	90	F:5′-ACGCTACGCTATACATGGACAC-3	60.3
			R:5′-AGGAGGAGACGATGAGGATGAG-3′	60.5
Ceramide Glucosyltransferase	*UGCG*	75	F:5′-TGCTCAGTACATTGCCGAAGA-3′	59.7
			R:5′-GTGGACATTGCAAACCTCCAA-3′	59.3
Sphingosine-1-phosphate phosphatase 1	*SGPP1*	137	F:5′-CTGGTGTTCTCTAGTTTGCCTAAG-3′	59.1
			R:5′-GGTTGAAGTTGTCAATCAGGTCC-3′	59.8
Beclin-1	*BECN1*	127	F: 5′-GGCTGAGAGACTGGATCAGG-3′	59.3
			R:5′-CTGCGTCTGGGCATAACG-3′	58.6
Microtubule-associated proteins 1A/1B	*LC3*	186	F:5′-AGCAGCATCCAACCAAAATC-3′	57
Light chain 3A			R:5′-TGTGTCCGTTCACCAACAG-3′	57.9
Lysosome-associated membrane glycoprotein	*LAMP*	195	F:5′-CTGCCTTTAAA GCTGCCAAC-3′	57.9
			R:5′-TGTTCTCGTCCAGCAGACAC-3′	60
Sequestosome-1	*P62*	86	F:5′-CAGAGAAGCCCATGGACAG-3′	57.5
			R:5′-AGGTGCCTTGTACCCACATC-3′	59.7
Fatty acid synthase	*FASN*	131	F:5′-TTCTACGGCTCCACGCTCTTCC-3′	64.3
			R:5′-GAAGAGTCTTCGTCAGCCAGGA-3′	61.7

qPCR primers: F, forward primer; R, reverse primer.

### 2.4 Western blotting examination

Proteins were extracted by a nuclear protein and cytoplasmic protein extraction kit (Servicebio, Ghent, Belgium). Bicinchoninic acid (BCA) protein concentration assay kit (Servicebio) was used to measure protein concentration. Western blotting analyses were performed as usual procedures. Rabbit anti-human FASN polyclonal antibody (FASN) (abmart, Shanghai, China; 1:1,000), rabbit anti-human Beclin1 polyclonal antibody (Beclin1) (abmart, 1:1,000), and rabbit anti-human LAMP polyclonal antibody (Beclin1) (abmart, 1:1,000) were used as the primary antibodies for CCs. Rabbit anti-human ASAH polyclonal antibody (29) (abcam, Cambridge, UK; 1:1,000), rabbit anti-human Beclin1 polyclonal antibody (Beclin1) (abmart, 1:1,000), and rabbit anti-human LAMP polyclonal antibody (Beclin1) (abmart, 1:1,000) were used as the primary antibodies for MGCs. Goat anti-mouse IgG conjugated with horseradish peroxidase (HRP) was used as a secondary antibody (abclonal, 1:3,000). The density of the target bands was analyzed by the Alpha software processing system (30).

### 2.5 Data analysis

The original data of LC-MS/MS were converted into.mzXML format by ProteoWizard, and then the XCMS program was used for peak alignment, retention time correction, and peak area extraction. Data were preprocessed by Pareto scaling, and multi-dimensional and one-dimensional statistical analyses were performed. Statistical analysis includes Student’s t-test and multiple variation analysis, and volcano maps were drawn by R software (23, 31). The data of qPCR were analyzed using GraphPad PRISM 9 software (GraphPad Software Version 9, La Jolla, CA, USA) and free software for data manipulation, calculation, and graphical display, and the mRNA expression level of the target gene was expressed as the mean ± standard deviation. The independent-samples t-test was used to assess statistical significance, and p < 0.05 was considered statistically significant.

## 3 Result

### 3.1 Demographic data

Demographic data from women with and without endometriosis are summarized in **Table 2**. There were no significant differences in age, body mass index (BMI), and the other baseline clinical and endocrine profiles in women in the endometriosis group compared with the control group except that the level of estradiol was lower in the endometriosis group (609.3 pmol/L) when compared with the control group (172 pmol/L). The fertilization rate was lower in the endometriosis group (98.28%) when compared with the normal group (88.81%).

**Table 2:** Clinical characteristics of study participants.

Parameters	Normal (N = 116)	Endometriosis (N = 43)	p-Value
Age (year)	32.16 ± 3.156	32.49 ± 3.269	0.559
weight (kg)	58.76 ± 9.949	58.94 ± 8.028	0.914
BMI (kg/m^2^)	22.52 ± 3.422	21.97 ± 2.864	0.357
FSH (mIU/ml)	8.007 ± 3.087	7.891 ± 2.972	0.832
LH (mIU/ml)	5.221 ± 2.608	5.854 ± 3.029	0.197
Total testosterone (nmol/L)	1.11 ± 2.475	0.8234 ± 0.4951	0.48
E2 pmol/L	609.3 ± 1683	172 ± 73.43	0.091
Number of oocytes retrieved	8.699 ± 5.918	9.095 ± 8.616	0.757
Number of mature oocytes	7.065 ± 5.054	7.857 ± 7.751	0.48
Number of fertilized oocytes	6.237 ± 4.904	6.571 ± 6.352	0.739
Fertilization rate	98.28 ± 109.3	88.81 ± 43.62	0.589
% Clinical pregnancy rate	33.72	32.35	

Data are means ± standard deviation.

BMI, body mass index; E2, estradiol; FSH, follicle-stimulating hormone; LH, luteinizing hormone; T, testosterone.

### 3.2 Metabolomic profiling of cumulus cells and mural granulosa cells in endometriosis and control women

**Figure 1:**
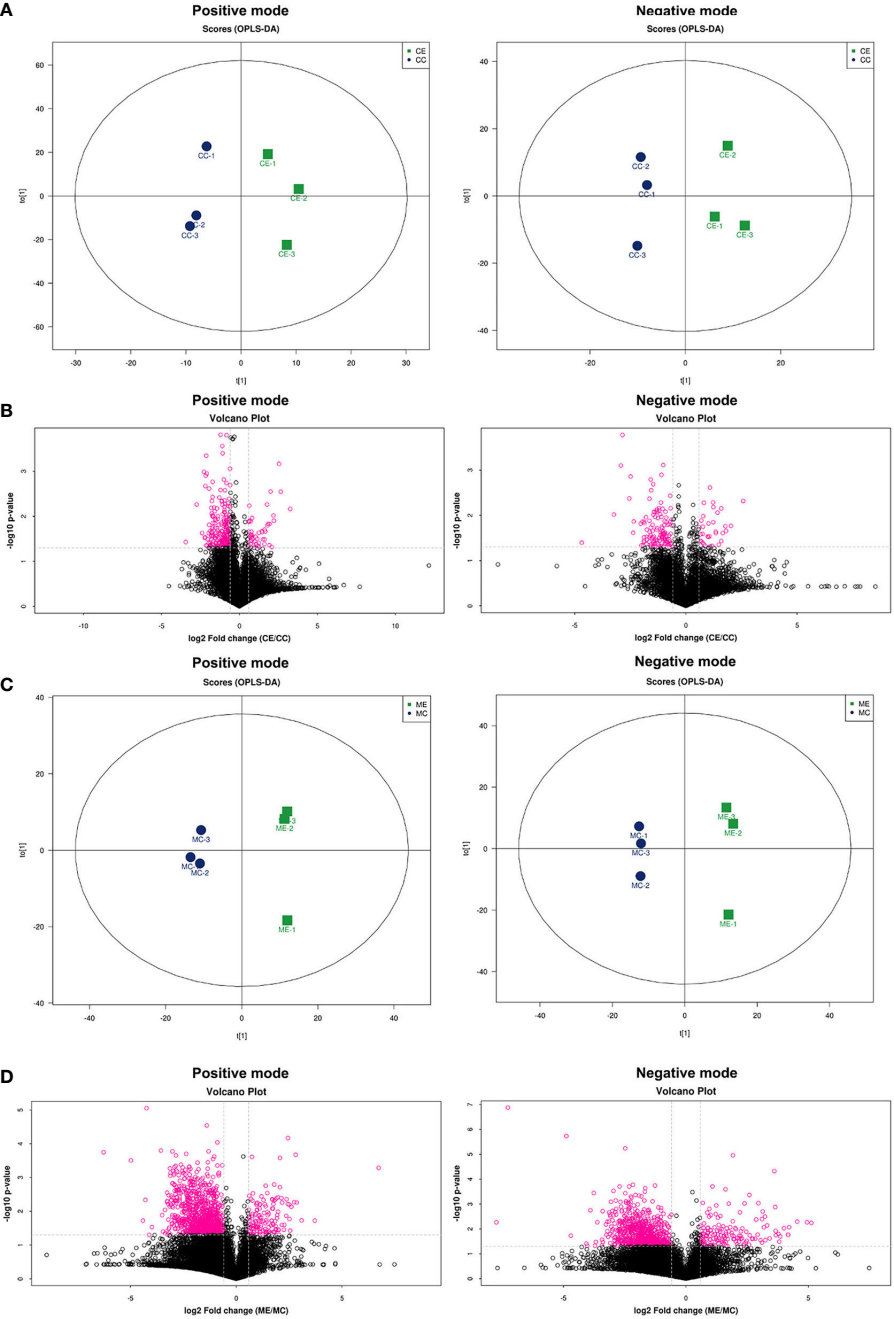
Metabolomic profiling of CCs and MGCs in endometriosis and control women by PLS-DA score plots and volcano plots. Score plots of principal component analysis of metabolome in the positive ion modes and the negative ion modes from CCs **(A)** and MGCs **(C)**. These plots display a clear separation between normal and endometriosis metabolomes. The circle (blue and green) around each sample group represents the 95% confidence intervals. Volcano plot of CCs **(B)** and MGCs **(D)** showing the statistical significance (y-axis) and fold change (x-axis) for the difference between the metabolome of normal and endometriosis. p <  0.05, fold change ≥ 1.5 in positive ion mode and negative ion mode. CCs, cumulus cells; MGCs, mural granulosa cells; PLS-DA, partial least squares discriminant analysis.

Partial least squares discriminant analysis (PLS-DA) score plots showed that supervised multivariate analysis revealed more obvious differences between the metabolites between endometriosis and control, indicating a significant difference in metabolic components in positive ion mode and negative ion mode both in CCs (**Figure 1A**) and in MGCs (**Figure 1C**). A volcano plot showed the difference in metabolites between the two kinds of cells, calculated from data for all tested substances. Cluster analysis of all the samples further revealed that the repeat results for endometriosis and controls clustered together, indicating a significant difference in metabolism both in CCs (**Figure 1B**) and in MGCs (**Figure 1D**).

### 3.3 Metabolomic analysis of cumulus cells in endometriosis and control women

#### 3.3.1 Heat map and metabolite–metabolite correlation analysis

**Figure 2:**
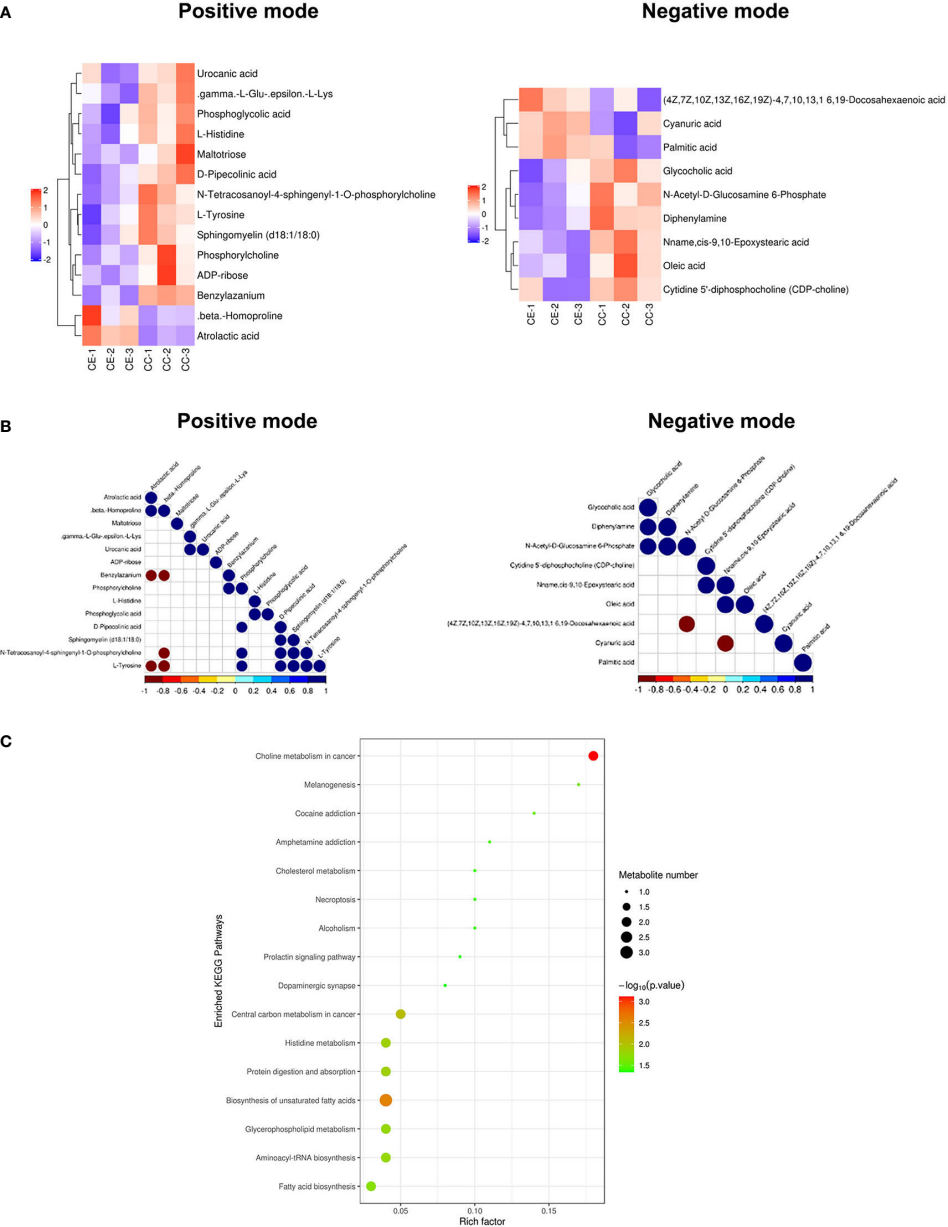
Metabolomic analysis of CCs in endometriosis and control women. Heat map of the changes in metabolites related to cumulus cells in endometriosis and control women. A heat map from cumulus cells in the positive ion modes and negative ion modes **(A)**. These plots display a clear separation between normal and endometriosis metabolomes. Data of heap map plots reveal unit-variance scaling. The blue color represents the trend of reduction, and red color represents an increasing trend. Metabolite–metabolite correlation analysis of cumulus cells in endometriosis and control women in the positive ion modes and negative ion modes **(B)**. Positive correlations are shown in blue, and negative correlations are shown in red. KEGG-enriched pathways changed between endometriosis patients and control **(C)**. CCs, cumulus cells; KEGG, Kyoto Encyclopedia of Genes and Genomes.

**Figure 3:**
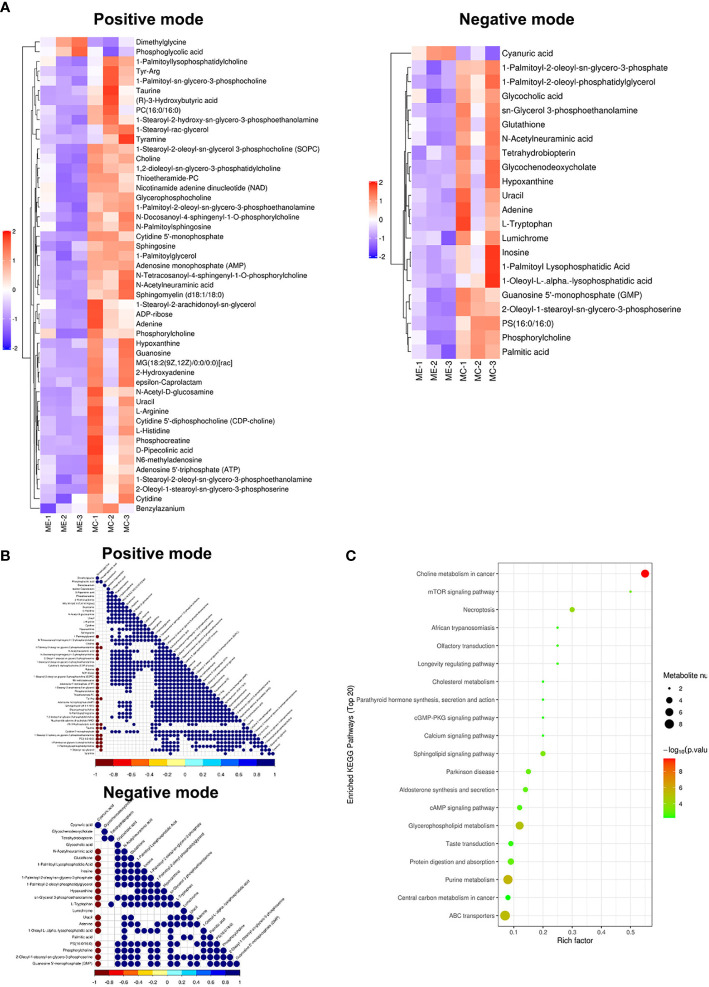
Metabolomic analysis of MGCs in endometriosis and control women. Heat map of the changes in metabolites related to MGCs in endometriosis and control women. A heat map from MGCs in the positive ion modes and negative ion modes **(A)**. These plots display a clear separation between normal and endometriosis metabolomes. Data of heap map plots reveal unit-variance scaling. The blue color represents the trend of reduction, and red color represents an increasing trend. Metabolite–metabolite correlation analysis of MGCs in endometriosis and control women in the positive ion modes and negative ion modes **(B)**. Positive correlations are shown in blue, and negative correlations are shown in red. KEGG-enriched pathways changed between endometriosis patients and control **(C)**. MGCs, mural granulosa cells; KEGG, Kyoto Encyclopedia of Genes and Genomes.

The results of the metabolomic analysis showed that there were differences in the metabolic profile of CCs and MGCs in endometriosis patients compared to controls. There were a total of 20,556 metabolite ion peaks: 11,784 in the positive ion mode and 8,772 in the negative ion mode. In CCs, 24 metabolites showed a difference in metabolite level between endometriosis and control, 14 different metabolites in positive ion mode, and 10 different metabolites in negative ion mode (**Figure 2A**). In MGCs, 71 metabolites showed a difference in metabolite level between endometriosis and control, 49 different metabolites in positive ion mode, and 22 different metabolites in negative ion mode (**Figure 3A**). The metabolites of variable importance in projection (VIP) >1 with multi-dimensional statistical analysis and p-value <0.05 with univariate statistical analysis as the significant differential metabolites were selected.

For preciseness, the role of cumulus cells on induced metabolites in endometriosis and multiple metabolites was screened. These metabolites were randomly divided into four different types including carbohydrate, amino acid, lipid, and other metabolites. There were differences in the metabolic profiles of CCs and MGCs between endometriosis and control. There were 3 metabolites upregulated in MGCs of endometriosis including, 1 lipid, 1 amino acid, and 1 other, whereas 68 metabolites were downregulated including 27 lipids, 8 amino acids, 4 carbohydrates, 6 energy, 9 nucleotides, and 9 nucleic acid pathways. All the detected metabolites were analyzed by heat mapping. In CCs, there were 5 metabolites upregulated in endometriosis, including 1 carbohydrate, 2 lipids, and 2 others, whereas 19 metabolites were downregulated including 7 lipids, 4 amino acids, 3 carbohydrates, 1 nucleic acid, and 4 other pathways. All the detected metabolites were analyzed by heat mapping. As shown in **Figure 2A**, the heat map indicated significantly increased levels of palmitic acid and docosahexaenoic acid (DHA) at 1.7859 and 1.8403, respectively, while a significantly decreased level of sphingomyelin at 0.445-fold change in CCs of endometriosis patients; in MGCs, there was a downregulation of sphingosine as 0.369-fold change (**Figure 3A**). Metabolite–metabolite correlations between the tissue of CCs and MGCs between endometriosis and control showed unique profiles. Metabolite–metabolite correlations showed significant correlation coefficients (p < 0.01) in CCs (**Figure 2B**) and in MGCs (**Figure 3B**). Moreover, palmitic acid, DHA, and sphingomyelin also played important roles in metabolite correlations in cumulus cells of endometriosis patients, while sphingosine showed played important roles in metabolite correlation of MGCs.

#### 3.3.2 Kyoto encyclopedia of genes and genomes enrichment

Our results showed that CCs and MGCs differentially expressed metabolites in the Kyoto Encyclopedia of Genes and Genomes (KEGG) pathway enrichment analysis between endometriosis patients and control. We used the qualitative difference in metabolite expression quantity of each sample. In hierarchical clustering, the same group of samples can appear in the same cluster through clustering. Meanwhile, metabolites clustered in the same cluster have similar expression patterns and may be in relatively close reaction steps in the metabolic process. **Figures 2C**, **3C** shows the results of the metabolite hierarchy clustering with significant differences in the sample group. The results showed that the choline metabolism in cancer and biosynthesis of unsaturated fatty acids were significantly changed between cumulus cells of endometriosis patients and control (**Figure 2C**), while the choline metabolism in cancer, glycerophospholipid metabolism, purine metabolism, and ABC transporters were significantly changed between MGCs of endometriosis patients and control (**Figure 3C**).

### 3.4 Gene and protein expression in cumulus cells and mural granulosa cells

**Figure 4:**
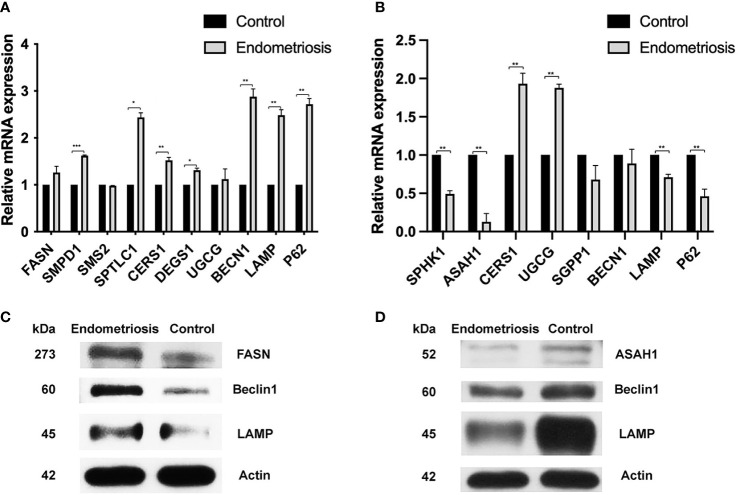
qRT-PCR expression analysis and protein expression of the ceramide synthesis and the autophagic pathway in CCs and MGCs compared between normal and endometriosis patients. **(A)** The expression levels of a gene involved in ceramide synthesis (CERS1, SPTL1, SMPD1, SMPD2, and SMPD3) and genes involved in autophagy (BECN1, LAMP, and P62) were analyzed in CCs compared between normal and endometriosis patients. **(B)** The expression levels of a gene involved in ceramide synthesis (SPHK1, ASAH1, CERS1, UGCG, and SGPP1) and genes involved in autophagy (BECN1, LAMP, and P62) were analyzed in MGCs compared between normal and endometriosis patients. Data (technical triplicates of three biological experiments) are reported as means ± standard error. *, **, *** indicate a statistically significant difference (p< 0.05, 0.01 and 0.001 respectively). Protein expression in CCs **(C)** and MGCs **(D)** was determined by Western blotting using antibodies against FASN, ASAH1, Beclin1, LAMP, and Actin. The actin band indicates equal loading of proteins. CCs, cumulus cells; MGCs, mural granulosa cells.

Concerning the alteration of palmitic acid and sphingomyelin in CCs of endometriosis patients compared to controls, we evaluated the level of gene involvement in ceramide synthesis and autophagic pathway. In detail, genes involved in the ceramide synthesis including Cers1, sptl1, and SMPD1 increased significantly (with p < 0.05) in cumulus cells of women with endometriosis when compared to normal (1.5-fold, 2.4-fold, and 1.6-fold, respectively) (**Figure 4**). Moreover, the autophagic gene expression including BECN1, LAMP, and P62 increased significantly (with p < 0.05) in CCs of endometriosis (2.8-fold, 2.4-fold, and 2.6-fold, respectively) but not in MGCs of endometriosis (**Figure 4A**). Our Western blotting results showed that the expression of FASN, Beclin1, and LAMP in CCs of the endometriosis group was higher than that of the control group (**Figure 4C**).

Concerning the alteration of sphingosine in MGCs of endometriosis patients compared to controls, we evaluated the level of gene involvement in sphingosine synthesis, S1P synthesis, ceramide synthesis, and autophagic pathway. In detail, genes involved in sphingosine synthesis including ASAH1 and genes involved in S1P synthesis including SPH1K decreased significantly (with p < 0.05) (0.1-fold and 0.48-fold, respectively), while genes involved in the ceramide synthesis including Cers1 increased significantly (with p < 0.05). In addition, UGCG mRNA levels in MGCs were significantly higher in endometriosis patients than in controls (1.88-fold). However, the autophagic gene expression including BECN1, LAMP, and PC3 decreased in MGCs of endometriosis (**Figure 4B**). Our Western blotting results showed that the expression of ASAH1, Beclin1, and LAMP in the CC of the endometriosis group was higher than that of the control group (**Figure 4D**).

## 4 Discussion

In endometriosis, changes in the follicular microenvironment of the COC lead to poor oocyte quality. As our results showed, the rate of fertilization and the level of estradiol in endometriosis had a tendency to be lower than in healthy women. However, the role of CCs and MGCs in endometriosis is still unclear. CCs around the oocyte play a critical role in supporting the oocyte maturation (32) and in steroidogenesis by promoting P450 aromatase activity for estrogen synthesis, which is important for follicular development and generating a competent oocyte to reach the mature metaphase II (MII) stage and fertilization (33). However, MGCs line the follicular wall and perform endocrine functions as well as assist follicular development (34). Therefore, it is possible that alteration of fatty acids in the COC, especially in CCs, influences the oocyte and fertilization. Adverse effects of endometriosis in the pathophysiology of the CCs have been extensively indicated including alteration in the cell cycle (29) and disruption of signaling pathways of CC growth and development (35, 36). CCs are in transcripts related to metabolism and cell proliferation in mice, whereas MGCs are rich in transcripts related to cell signaling and differentiation. In mice, CCs are enhanced in transcripts related to metabolism and cell proliferation, whereas MGCs are enhanced in transcripts related to cell signaling and differentiation (37). Moreover, CCs were shown to be high in metabolites linked to cholesterol transport and estradiol generation in human preovulatory follicles, while MGCs were enriched in metabolites associated with anti-apoptosis (38). Additionally, previous studies demonstrated that many types of endometriosis also may negatively affect CCs steroidogenesis by reducing the level of P450 aromatase, increasing ROS, and inducing apoptosis (4, 39). A previous report has shown that endometriosis impaired CC steroidogenesis, leading to an imbalance in estrogen synthesis (40). Interestingly, cell death of the follicular cells can be induced through oxidative stress, hyper-androgenemia, and disturbance of gonadotropin hormone. Endometriosis impairs the cell cycle in granulosa cells (29). Recently, many reports indicated that estrogen receptor (ER) stress induction results in alteration of cumulus–oocyte complex (41), activation of ovarian fibrosis (42), and apoptosis of follicular cells (43). Moreover, a previous report found that CCs from endometriosis patients reveal senescence through ER stress-associated endometriosis-associated infertility and senescence phenotype (44).

Sphingolipid metabolism is important for maintaining normal follicular and oocyte maturation through modulating energy metabolism, cell proliferation, apoptosis, and steroid hormone synthesis (13). Previous studies reported that sphingolipid metabolism in endometriosis showed alteration in the serum, plasma, peritoneal fluid, follicular fluid, and endometrial tissue (13, 23, 45–47). However, no information is reported about CCs and MGCs correlated between sphingolipid metabolism and autophagic pathway in endometriosis. Our study focuses on the role of these follicular cells in terms of metabolism and integrated biochemical studies. Interestingly, our results demonstrated that the metabolites related to palmitic acid, sphingolipid metabolism, and autophagic cell death were enriched in only CCs, indicating that sphingolipid metabolism plays a critical role in follicle and oocyte growth. In the literature, previous evidence showed that sphingolipid metabolism regulated cell death at the molecular level. In this study, we used a metabolomic assay to evaluate the sphingolipid metabolism of CCs and MGCs in endometriosis systemically. Our results represented the alteration of the biomolecules presented in CCs and MGCs in endometriosis patients. The metabolic profiling of CCs has evidenced that the level of palmitic acid was increased in CCs but not in MGCs according to upregulated FASN gene expression in CCs. Therefore, the metabolite results suggested that palmitic acid may play an essential role in CCs and MGCs of endometriosis. Several studies suggested that elevation of palmitic acid leads to inflammation in several tissues during pathophysiologic progression (48, 49). Additionally, recent studies demonstrated that the accumulation of palmitic acid and its downstream metabolism leads to inflammation and cell death (50, 51). Y. M. Mu’s team showed that saturated FFAs, palmitic acid, and stearic acid induce apoptosis in human granulosa cells *in vitro* (17). In the COC, oocytes seem to be more sensitive to fatty acid stress. Moreover, several reports suggested elevation of fatty acids in the blood, and follicular fluid affected oocyte maturation (14, 15, 52–54). Previous reports on obesity elevated palmitic acid can cause insulin resistance (IR) and impairment of glucose metabolism in ovarian GCs (55). This evidence supports that palmitic acid is one of the key molecules in lipid metabolism. In addition, the alteration of sphingolipidomic data in serum, peritoneal fluid, and endometrial tissue in endometriosis patients reveals the critical role of lipid metabolism in pathological progression (13). Interestingly, we suggested that sphingolipid metabolism can be used by cumulus cells, which mediate its effect on oocyte maturation.

The key molecules in sphingolipid metabolism are ceramide and sphingosine-1-phosphate (S1P), which play a critical role in survival and cell death. Ceramide is a bio-effector molecule that mediates cell death, whereas S1P induces cell proliferation (56). Ceramide is a central molecule of sphingolipid metabolism and serves as the precursor for several sphingolipids including sphingomyelin (SM) and Cer-1-phosphate and glucosylceramide (GlcCer).

Recent studies also demonstrated that elevation and accumulation of GlcCer are associated with many human diseases including Gaucher’s disease, polycystic kidney disease, diabetes, and endometriosis, leading to overproliferation (57). Both *in vitro* and *in vivo* studies indicated that GlcCers inhibited GlcCer synthase (GCS) through several processes leading to cell death (58–60). Moreover, endometriosis patients reveal an association between GCS level and serum and PF GlcCer accumulation, indicating abnormalities in endometrial proliferation. Interestingly, an increasing level of Cer would have induced apoptosis in endometrial cells (61, 62). According to our results, the elevation of genes involved with the ceramide synthesis (Cers1, sptl1, and SMPD1) in CCs associated with elevated palmitic acid in metabolomic analysis supports that sphingolipid metabolism may be associated with the pathophysiology of endometriosis (13). However, sphingolipid metabolites in MGCs found that low levels of palmitic acids, sphingomyelin, and sphingosine are associated with the low level of the ceramide synthesis gene. Our studies indicated that sphingolipid metabolism is mediated pathway through regulating cell survival and cell death.

Autophagy is one of the types of cell death that modulate cellular homeostasis by supplying the cell with energy and metabolites and preventing oxidative stress to maintain normal cellular function under stress conditions (63, 64). A previous report demonstrated that the key molecule of sphingolipid metabolism, ceramide (Cer), induced autophagic cell death (65). Our results also revealed that cluster genes and proteins in autophagy (BECN1 and LAMP2) significantly increased in association with an increased level of palmitic acid and the ceramide synthesis gene in CCs under the pathological condition of endometriosis. This evidence indicated that CCs may undergo cell death, which may directly affect the oocyte quality. This evidence indicates that sphingolipid metabolism in surrounding cumulus cells plays an important role in the maturation of oocytes and steroidogenesis. However, in MGCs, this cluster of genes decreased in endometriosis, which correlated with the role of MGCs in the angiogenesis compartment of follicular cells.

A recent study demonstrates the association between apoptosis and survival molecules in cumulus cells can be used as a marker for estimating oocyte quality (66). Interestingly, our study indicated that the poor oocyte quality in patients with endometriosis is related to a higher level of sphingolipid metabolism correlated with the increase of autophagic gene and protein levels in the CCs but not in the MGCs. A balance in sphingolipid metabolism in CCs is associated with oocyte maturation and further development of an embryo.

The number of cumulus cells closely associated with energy sufficiency cell proliferation directly affects oocyte development. The cell depends on apoptosis and autophagic signaling pathways; therefore, saturating FFA metabolites may potentially promote cell death (17). We can suggest that this procedure could be integrated through biochemical studies in the follicular cells. It is better known that oocyte quality depends on the follicular microenvironment, and adequate bidirectional signaling between COC is important for both oocyte and cumulus cell competence acquisition (67). The most relevant and significant data of metabolites we obtained on the CCs and MGCs in endometriosis patients (autophagic genes and proteins decrease linked to the increase of lipid levels) could then be used to help to identify oocytes with higher capacity for development and fertilization to enhance the possibility of success of *in vitro fertilization* (IVF) and improving the probability of pregnancy in endometriosis patients.

The metabolic characterization of CCs and MGCs to integrate the multiple approaches from biochemical analysis to gene analysis in association with pathogenesis and disease progression has gained more value in understanding endometriosis. Our work demonstrates how integrating the different approaches of the ‘omic’ sciences from metabolomics to gene expression can help in better understanding the mechanisms of endometriosis progression and can lead to improvement of diagnosis. The advantage of systems biology is the integration of proteomics, transcriptomics, and metabolomic information to obtain a better understanding mechanism of human pathophysiology. Moreover, insight into the differences between the metabolic profile of CCs and MGCs may lead to the discovery of a new biomarker for estimating the quality of oocytes in endometriosis patients. It is possible that CCs and MGCs associated with competent oocytes have fulfilled their role of sustaining oocyte development, and their response to metabolic alteration is under consideration. Taken together, these findings may optimize the development of novel methods to improve oocyte selection strategies in assisted reproductive technology (ART) protocols and shed light on the molecular mechanisms governing oocyte–CC–MGC cross-talk. The functional roles of those metabolites in follicles related to oocyte growth, maturation, and subsequent embryonic developmental need to be further explored and studied.

## 5 Conclusion

**Figure 5:**
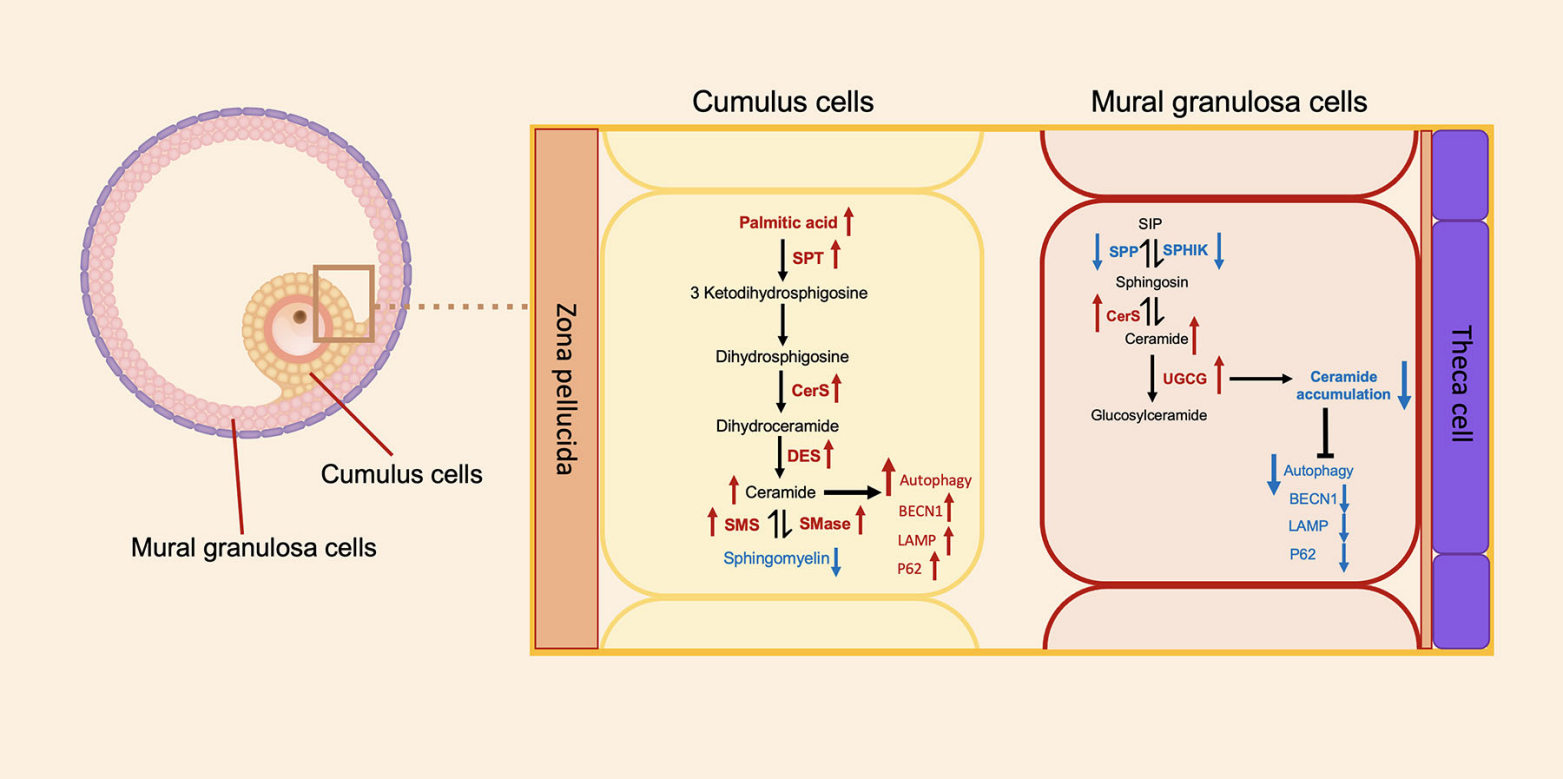
Schematic representation of the metabolomics of sphingolipids and autophagic gene expression between the cumulus cells and the mural granulosa cells. Direction of arrows represents the direction of possible activity of sphingolipid pathways. Red colors of arrows represent increased level of metabolite or gene, and blue colors of arrows represent decreased level of metabolite or gene. SPT, serine palmitoyltransferase; CerS, ceramide synthase; DES, dihydroceramide desaturase; SMS, sphingomyelin synthase; SMase, sphingomyelinase; SPP, sphingosine-1-phosphate phosphatase; SPHIK, sphingosine kinase; UGCG, UDP-glucose ceramide glucosyltransferase; BECN1, Beclin1; LAMP, lysosome-associated membrane protein.

The results indicate that CCs and MGCs reveal differences in terms of metabolites. CCs show the alteration of lipid metabolism correlated with autophagic cell death, while MGCs show no alteration, as shown in **Figure 5**. Our finding provides new insight into follicular cells in endometriosis.

## Data availability statement

The original contributions presented in the study are included in the article/supplementary materials. Further inquiries can be directed to the corresponding author.

## Ethics statement

This study was reviewed and approved by the Medical and Life Science Ethics Committee of Tongji University (approval No. 2017yxy001). The patients/participants provided their written informed consent to participate in this study.

## Author contributions

R-CC: study design, manuscript writing, and critical discussion. BT and E-MG.: study design, experiment execution, data analysis, manuscript writing, and critical discussion. KG: experiment execution, manuscript writing, and discussion. Y-BL, LW, and XD: experiment execution and manuscript review. All authors contributed to the article and approved the submitted version.

## Funding

This work was supported by the National Key R&D Program of China (No. 2017YFC1002003) and the Ministry of Science and Technology of China (No. 2017YFC1001601).

## Conflict of interest

The authors declare that the research was conducted in the absence of any commercial or financial relationships that could be construed as a potential conflict of interest.

## Publisher’s note

All claims expressed in this article are solely those of the authors and do not necessarily represent those of their affiliated organizations, or those of the publisher, the editors and the reviewers. Any product that may be evaluated in this article, or claim that may be made by its manufacturer, is not guaranteed or endorsed by the publisher.
